# Establishing a new human pneumococcal standard reference serum, MPRSS-01

**DOI:** 10.1128/msphere.00404-24

**Published:** 2025-01-07

**Authors:** Joseph M. Antonello, Rocio D. Murphy, Chitrananda Abeygunawardana, Juliana C. Malinverni, Rajam Gowrisankar, Tina Green, Rebecca Greway, Jenna Schmauch, Adrienne Howlett, Cyrille J. Bonhomme, Katrina M. Nolan

**Affiliations:** 1 Merck & Co., Inc., Rahway, New Jersey, USA; 2 PPD Vaccine Sciences, part of Thermo Fisher Scientific, Richmond, Virginia, USA; The University of Texas Medical Branch at Galveston, Galveston, Texas, USA

**Keywords:** MPRSS-01, 007sp, pneumococcal vaccine standardization, pneumococcal standard reference

## Abstract

Measuring the immunogenicity of pneumococcal vaccines involves the use of immunoassays to measure serotype-specific immunoglobulin G (IgG) antibody levels post-vaccination with the current *Streptococcus pneumoniae* human reference serum standard (007sp) for anti-pneumococcal capsule antibodies. Development of new pneumococcal conjugate vaccines (PCVs) with additional serotypes not in 007sp (e.g., V116, a 21-valent PCV) requires a new reference serum. Antibody concentrations to 33 pneumococcal serotypes were assigned in a new Merck Pneumococcal Reference Serum Standard (MPRSS-01; Merck Pneumococcal Reference Serum Standard) using the pneumococcal electrochemiluminescence assay. MPRSS-01 was generated by pooling high-titer serum samples from adults immunized with either 23-valent pneumococcal polysaccharide vaccine (PPSV23) or V116. For the 24 serotypes with established IgG concentrations, the corresponding antibody concentrations in MPRSS-01 were assigned via direct calibration to 007sp, while a cross-standardization approach was used for the nine novel serotypes. Serotype 7F was initially chosen as the reference calibrator for cross-standardization due to parallelism across dilution–response curves demonstrated across all 33 serotypes, and an evaluation of the single-calibrator approach was conducted for the 24 serotypes. Potential systematic bias from using a unique serotype for calibration was identified and addressed by further adjusting the estimated IgG concentrations of the nine novel serotypes. Using the final MPRSS-01 antibody concentration assignments, and the calibration factor relating MPRSS-01 to 007sp, antibody concentration assignments for 007sp were provided for the nine novel serotypes. This proposal was accepted by the Center for Biologics Evaluation and Research (CBER), enabling V116 to bridge old and new human pneumococcal reference sera.

**IMPORTANCE:**

Immunogenicity of pneumococcal vaccines is measured using post-vaccination serotype-specific immunoglobulin G (IgG) antibodies in serum using enzyme-linked immunoassays with the 007sp reference serum containing serotype-specific IgG for 24 pneumococcal serotypes. With the development of next-generation PCVs, a new *S. pneumoniae* reference serum standard was needed to include serotypes beyond the existing 24 in 007sp. In this study, antibody concentrations to 33 pneumococcal serotypes were assigned in a new Merck Pneumococcal Reference Serum Standard (MPRSS-01) using the pneumococcal electrochemiluminescence assay, enabling V116 to maintain the link to the historical human pneumococcal standard reference serum while utilizing the new human pneumococcal reference serum.

## INTRODUCTION

Evaluating immunogenicity in pneumococcal vaccine development involves measuring serotype-specific anti-pneumococcal serum immunoglobulin G (IgG) levels after vaccination using an appropriate antibody capture assay, such as the enzyme-linked immunosorbent assay (ELISA). From 1990 to 2013, Lot 89SF was the human anti-pneumococcal standard reference serum pool used in pneumococcal ELISAs (1). Lot 89SF was prepared from adult sera post-immunization with 23-valent pneumococcal polysaccharide vaccine (PPSV23). Using ELISA methodology and human standard reference serum US National Reference Preparation for Human Serum Proteins (USNRP) IS 1644, serotype-specific weight-based values for IgG, immunoglobulin A (IgA), immunoglobulin M (IgM), and total immunoglobulin (Ig) for serotypes 1, 3, 4, 5, 6B, 7F, 9V, 14, 18C, 19F, and 23F were derived for Lot 89SF by Quataert et al. (2). In 2004, weight-based IgG, IgA, IgM, and total Ig antibody assignments were made for the additional serotypes in PPSV23 (2, 6A, 8, 9N, 10A, 11A, 12F, 15B, 17F, 19A, 20, 22F, and 33F), as well as for cell wall polysaccharide (CWPS). The additional 14 assignments were determined using an equivalence of absorbance method with an anti-pneumococcal polysaccharide (PnPs) serotype 6B reference ELISA. Owing to dwindling supplies of 89SF, a new reference standard, 007sp, was developed and evaluated in 2011 (1). Five independent laboratories using the World Health Organization (WHO) reference ELISA participated in bridging the serotype-specific IgG assignments for 89SF to 007sp to establish equivalent reference values for 13 pneumococcal capsular serotypes (1, 3, 4, 5, 6A, 6B, 7F, 9V, 14, 18C, 19A, 19F, and 23F). With the requirement to evaluate PPSV23 and pneumococcal conjugate vaccines (PCVs) that may be developed in the future, serotype-specific IgG assignments to 007sp were subsequently obtained for serotypes 8, 10A, 11A, 12F, 15B, 22F, and 33F (3), and for serotypes 2, 9N, 17F, and 20A (4). More recently, with the 21-valent PCV, V116, developed by Merck & Co., Inc., Rahway, NJ, USA, which is specifically designed for the adult population (5), and the potential development of future PCVs containing serotypes beyond the 24 assigned in 007sp, a new *Streptococcus pneumoniae* reference serum standard was needed. This paper describes the efforts taken to establish the serotype-specific IgG concentrations for a new reference standard, Merck Pneumococcal Reference Serum Standard (MPRSS-01), for the 24 serotypes having a 007sp-established IgG concentration and nine novel serotypes in V116 not having a 007sp-established IgG concentration.

## MATERIALS AND METHODS

### Serum collection

007sp was generated under a US Food and Drug Administration (FDA)-approved clinical protocol, in which 278 adult volunteers were immunized with the 23-valent unconjugated polysaccharide vaccine PPSV23. A unit of blood was obtained two times from each immunized subject within 120 days following immunization. Pooled serum was prepared from the plasma of 262 individuals, filled at 6 mL per vial into 15,333 vials and lyophilized.

MPRSS-01 was generated from future biomedical research-consented sera from adults immunized with either PPSV23 or the 21-valent PCV, V116 (6). All serum samples were appropriately consented by the donors to reflect the current requirements for human studies. The sera were collected 30 days post-immunization. MPRSS-01 comprises approximately 50 mL of pooled serum from 17 adults, eight immunized with PPSV23 and nine immunized with V116. The serum was pooled in 3 mL equivalent volumes across the 17 participants and sub-aliquoted. No preservatives were added, and the serum was stored at ≤−60° C. While MPRSS-01 is nearing depletion, a new and substantially larger pool (20 times the volume of MPRSS-01) of reference serum, MPRSS-02, has been created and calibrated against MPRSS-01 for future clinical testing.

### Laboratory methods

A proprietary bioanalytical method for the detection of antibodies to 15 PnPs serotypes in human serum has been previously developed and validated for the 15-valent PCV, V114 (VAXNEUVANCE, Merck Sharp & Dohme LLC, a subsidiary of Merck & Co., Inc., Rahway, NJ, USA). The pneumococcal electrochemiluminescence (Pn ECL) assay (7) is based on the Meso Scale Discovery (MSD) ECL technology, which uses multi-spot microtiter plates fitted with a series of electrodes associated with the bottom of each well. Using an MSD plate imager, an electrical current is placed across the plate-associated electrodes. The result is a series of electrically induced oxidation–reduction reactions involving ruthenium and tripropylamine that lead to luminescent signal.

The Pn ECL assay was developed using CWPS, PnPs 25 (corresponding to serotype 25F per Danish nomenclature), and PnPs 72 (corresponding to serotype 45 per Danish nomenclature) for pre-adsorption of samples, standard, and controls to improve the specificity of the pneumococcal serotypes in the vaccine. Antibody concentrations were determined in an indirect binding format, in which serum sample IgG antibodies bind to PnPs on the MSD plate. Subsequently, Sulfo-Tag-labeled anti-IgG total antibodies bind to the serum sample antibodies. Test sample antibody concentration was determined via back-calculation of the ECL response against the concentration–ECL response curve of the reference standard tested on the same plate. The V116 Pn ECL assay was developed and validated similarly to the V114 method (8), with three MSD panels to cover 24 serotypes, including the nine V116-novel serotypes not having a 007sp-established IgG concentration.

**Fig 1:**
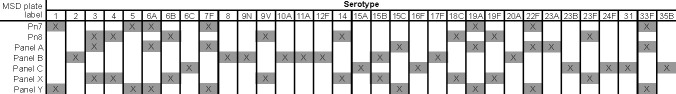
Pn ECL assay MSD plate constructs. X denotes the pneumococcal polysaccharide types that are spotted on the MSD plate. MSD, Meso Scale Discovery; Pn ECL, pneumococcal electrochemiluminescence.

The MPRSS-01 concentration assignments for the 33 serotypes evaluated in this paper utilized seven different MSD plate configurations comprising 7–9 serotypes each. The seven different groupings of serotypes were created to separate potentially cross-reactive serotypes. The serotypes evaluated within each of the seven MSD plate configurations are indicated in Fig. 1. As shown in Fig. 1, there is overlap of certain serotypes across the seven plate configurations. In cases in which the same serotype was tested in more than one plate configuration, the results from each configuration were included in the calibration assessment.

### Statistical methods

All pairwise dilution–response reference serum curves were simultaneously fit using the four-parameter logistic function following the methods of DeLean et al. (9) and O’Connell et al. (10). The reference serum curves decrease sigmoidally from a maximum level of response to a minimum level of response as a function of increasing dilution. Parallel-line analysis methodology was used to estimate the calibration factor relating the pairwise dilution–response curves. The adequacy of restricting the paired dilution series to share a common slope was assessed for each serotype via the ratio of the slope estimates from an individual slopes model of the form:


$$y_{i}=D+\left(A-D\right)\left[\left(\frac{I_{1}}{1+{\left(\frac{x_{i}}{C_{1}}\right)}^{-B_{1}}}\right)+\left(\frac{I_{2}}{1+{\left(\frac{x_{i}}{C_{2}}\right)}^{-B_{2}}}\right)\right]+e_{i},\;\;0<x_{i}<\infty$$

in which $y_{i}$ denotes the observed response at the *i*^th^ dilution $x_{i}$; *A* and *D* are the asymptotic maximum and minimum parameters, respectively; $B_{1}$ and $B_{2}$ are the slope parameters for the two curves; $C_{1}$ and $C_{2}$ are the dilutions corresponding to the 50% response for the curves; $I_{1}$ and $I_{2}$ are indicator variables that take on the value 0 or 1 depending on whether the response is a Curve 1 or Curve 2 measure; and $e_{i}$ represents the differences between the observed and fitted responses at the *i*^th^ dilution and are independent with mean 0 and variance proportional to the square of the expected level of response. The model fits were carried out using the *NLIN* procedure in SAS version 9.4 (the data analysis for this paper was generated using SAS software, SAS Institute Inc., Cary, NC, USA). Final parameter estimates were the values that minimized the weighted residual sum of squares, with the weights being inversely proportional to the square of the predicted response, thereby giving less weight to the less precise measurements and more weight to the more precise measurements when estimating the parameters. Parallelism assessments followed the equivalence testing approach of Hauck et al. (11) by computing the slope ratio and its approximate variance, which was obtained using the variance estimates of the model parameters provided by SAS software and application of the delta method. An overall estimate of the slope ratio across the runs for each curve pairing was the average of the individual ratios weighted by the inverse of their corresponding variance estimates. Details of the calculations are provided in the Appendix. Each of the pairwise curves under comparison was deemed sufficiently parallel, given that each slope ratio was close to 1.0, ranging between 0.90 and 1.11; and the bounds of the 95% confidence interval (95% CI) for each slope ratio fell between 0.91 and 1.05. Given that the dilution–response curves were found to be acceptably parallel in every instance, the pairwise dilution–response curves were simultaneously refit using the four-parameter logistic function given above, but with a common slope parameter B in place of $B_{1}$ and $B_{2}$. The calibration factor was the ratio of the $C_{1}$ to $C_{2}$ estimates from the common slope model. Details of the calculations for the overall calibration factor and its 95% CI for each curve pairing follow those provided in the Appendix for the slope ratio, but with ${\hat{C}}_{1j}$ and ${\hat{C}}_{2j}$ replacing ${\hat{B}}_{1j}$ and ${\hat{B}}_{2j}$ in the calculations.

### Experimental design

**Fig 2:**
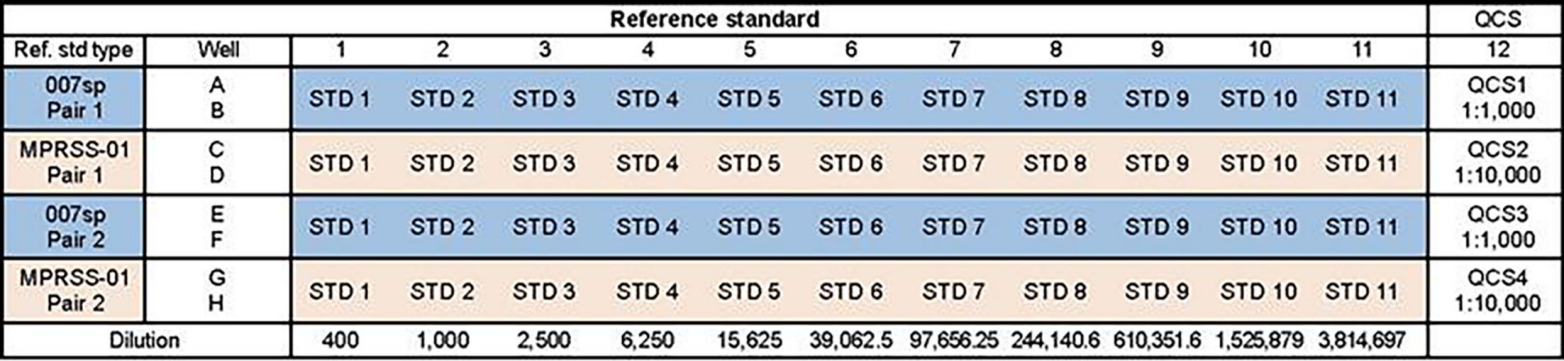
Sample plate layout for the MPRSS-01 calibration experiment. QCS1 and QCS3 were tested at the 1:1,000 dilution. QCS2 and QCS4 were tested at the 1:10,000 dilution. QCS, quality control sample; STD, standard.

The calibration testing comprised 12 assay runs carried out across 2 weeks by two analysts. Each assay run contained five MSD plates, the three V116 plates [Panels A (serotypes 3, 6A, 7F, 15C, 19A, 22F, 23A, and 33F), B (serotypes 2, 8, 9N, 10A, 11A, 12F, 15B, 17F, and 20A), and C (serotypes 6C, 15A, 16F, 23B, 24F, 31, and 35B)] and either the two V114 plates [Panels Pn7 (serotypes 1, 5, 6A, 7F, 19A, 22F, and 33F) and Pn8 (serotypes 3, 4, 6B, 9V, 14, 18C, 19F, and 23F)] or the Panel X (serotypes 3, 4, 6B, 9V, 14, 15B, 18C, 19F, and 23F) and Y (serotypes 1, 5, 6A, 7F, 15C, 19A, 22F, and 33F) configured plates. The inclusion of Panels 7 and 8 or Panels X and Y within a run allowed for the evaluation of all 33 serotypes within each run, as both Panels 7 and 8 and Panels X and Y contain the nine evaluated serotypes not contained in Panels A, B, and C. Each plate contained two independent preparations of the 007sp reference standard and two independent preparations of MPRSS-01 reference standard for a total of four independent preparations on each MSD plate. The MPRSS-01 and 007sp human sera reference standards were both analyzed at an initial dilution of 1:400 in serum diluent followed by 10 2.5-fold dilutions for a final dilution series of 1:400 to 1:3,814,697 prepared in singlet and transferred in replicate. In addition, four quality control samples (QCS) were tested in column 12 included in each plate. A sample plate layout is provided in Fig. 2.

## RESULTS

### Assignment of antibody concentrations to MPRSS-01 reference standard relative to 007sp reference standard

For each serotype having an established IgG antibody concentration for 007sp, the corresponding antibody concentration in MPRSS-01 was assigned via the direct calibration of MPRSS-01 to 007sp in the Pn ECL. A similar approach to assign antibody concentrations to 007sp was used by Goldblatt et al. (3, 4) in directly calibrating 007sp to human reference serum Lot 89SF, the original human anti-pneumococcal reference standard.

Parallel-line analysis using the four-parameter logistic regression function was used to estimate the calibration factor relating the dilution–response curve of the MPRSS-01 reference standard to the dilution–response curve of the 007sp reference standard. The adequacy of restricting the two-dilution series to share a common slope was assessed for each serotype. A slope ratio close to 1.0 indicates that the dilution series being compared have similar slopes, a desirable condition for calibration by parallel-line analysis. A slope ratio different from 1.0 means that the new reference standard cannot achieve concentration assignments equivalent to that of the current reference standard throughout the quantifiable range. For the Pn ECL assays, MPRSS-01 was deemed to be acceptably parallel to 007sp if the slope ratio was between 0.90 and 1.11 (greater detail on the utilized acceptance range is provided in the Discussion section). The estimated slope ratio for each serotype is provided in Table 1. The dilution–response curves between MPRSS-01 and 007sp were considered acceptably parallel for each of the 24 serotypes having a Goldblatt assignment, as the slope ratios ranged from 0.95 (serotype 5) to 1.02 (serotype 3). Parallelism of the dilution–response curves between MPRSS-01 and 007sp was also considered acceptable for each of the nine serotypes without a Goldblatt assignment, as those slope ratios ranged from 0.92 (serotype 6C) to 1.04 (serotype 23A).

**TABLE 1:** Serotype-matched calibration between MRPSS-01 and 007sp^*a*^

Serotype	*N*	Average slope		Slope ratio (MPRSS-01/007sp)		Calibration factor (MPRSS-01/007sp)		007sp-assigned conc (µg/mL)	MPRSS-01 conc based on 007sp (µg/mL)	
		MPRSS-01	007sp	Estimate	95% CI	Estimate	95% CI		Estimate	95% CI
1	24	1.03	1.05	0.98	0.97–0.98	0.67	0.65–0.69	8.50	**5.69**	5.57–5.82
2	23	1.04	1.06	0.97	0.97–0.98	0.99	0.97–1.02	24.63	**24.46**	23.87–25.07
3	47	1.07	1.05	1.02	1.01–1.02	1.71	1.66–1.76	1.45	**2.48**	2.41–2.56
4	24	1.06	1.07	0.99	0.98–0.99	0.59	0.58–0.61	3.33	**1.97**	1.92–2.02
5	24	1.06	1.11	0.95	0.95–0.96	0.35	0.34–0.36	7.51	**2.63**	2.55–2.70
6A	47	1.08	1.12	0.97	0.96–0.97	3.44	3.38–3.51	3.93	**13.54**	13.28–13.80
6B	24	1.11	1.10	1.01	1.00–1.01	1.05	1.03–1.08	9.05	**9.54**	9.33–9.76
6C	23	1.03	1.12	0.92	0.91–0.93	5.62	5.43–5.81	ND	ND	ND
7F	47	1.04	1.07	0.98	0.97–0.98	2.44	2.41–2.47	8.30	**20.24**	19.98–20.50
8	23	1.02	1.04	0.99	0.98–0.99	2.26	2.22–2.29	14.24	**32.13**	31.64–32.62
9N	23	1.06	1.09	0.97	0.96–0.97	1.68	1.65–1.71	7.03	**11.80**	11.59–12.02
9V	24	1.09	1.09	1.00	1.00–1.01	1.08	1.06–1.11	6.44	**6.96**	6.80–7.12
10A	23	1.07	1.07	0.99	0.99–1.00	3.47	3.42–3.52	12.98	**45.00**	44.35–45.67
11A	23	1.08	1.08	1.00	0.99–1.00	3.60	3.53–3.67	5.08	**18.28**	17.93–18.63
12F	23	1.02	1.03	0.99	0.99–1.00	6.06	5.96–6.15	2.21	**13.39**	13.18–13.60
14	24	1.03	1.05	0.99	0.98–0.99	1.53	1.49–1.57	37.99	**58.21**	56.75–59.72
15A	23	1.07	1.06	1.01	1.01–1.02	8.75	8.58–8.92	ND	ND	ND
15B	35	1.06	1.06	1.00	1.00–1.01	2.92	2.87–2.97	16.94	**49.45**	48.54–50.38
15C	35	1.06	1.06	1.00	1.00–1.01	4.24	4.17–4.31	ND	ND	ND
16F	23	1.16	1.13	1.02	1.01–1.03	4.18	4.09–4.26	ND	ND	ND
17F	23	1.05	1.06	0.99	0.98–1.00	4.66	4.56–4.76	8.51	**39.64**	38.80–40.50
18C	24	1.04	1.06	0.98	0.97–0.98	0.66	0.64–0.70	7.30	**4.85**	4.64–5.08
19A	47	1.07	1.07	1.00	1.00–1.00	1.38	1.36–1.40	13.87	**19.16**	18.87–19.47
19F	24	1.06	1.07	0.99	0.99–1.00	0.88	0.86–0.90	14.61	**12.83**	12.59–13.09
20A	23	1.05	1.06	0.98	0.98–0.99	4.97	4.87–5.06	10.47	**51.99**	51.04–52.95
22F	47	1.08	1.07	1.01	1.00–1.01	2.63	2.59–2.68	9.50	**25.03**	24.61–25.45
23A	23	1.12	1.07	1.04	1.03–1.05	6.32	6.21–6.44	ND	ND	ND
23B	23	1.09	1.09	0.99	0.98–1.00	4.89	4.74–5.04	ND	ND	ND
23F	24	1.07	1.08	0.99	0.99–1.00	1.79	1.70–1.88	5.95	**10.64**	10.13–11.18
24F	23	1.04	1.05	1.00	0.99–1.00	25.88	25.43–26.34	ND	ND	ND
31	23	1.11	1.10	1.01	1.00–1.01	6.99	6.84–7.15	ND	ND	ND
33F	47	1.06	1.07	0.99	0.98–0.99	3.32	3.26–3.37	10.66	**35.34**	34.75–35.95
35B	23	1.07	1.10	0.97	0.97–0.98	6.23	6.04–6.43	ND	ND	ND

^*a*^ Grey shading indicates the serotypes without previously published concentration assignments for 007sp. Bolded values indicate MPRSS-01 final concentration assignments for the 24 serotypes having a Goldblatt-assigned 007sp antibody concentration. The slope ratio estimate and its 95% CI were derived from the individual slopes model while the calibration factor and MPRSS-01 antibody concentration estimates were derived from the common slope model. International assignment does not exist. CI, confidence interval; Conc, concentration; ND, not determined.

**Fig 3:**
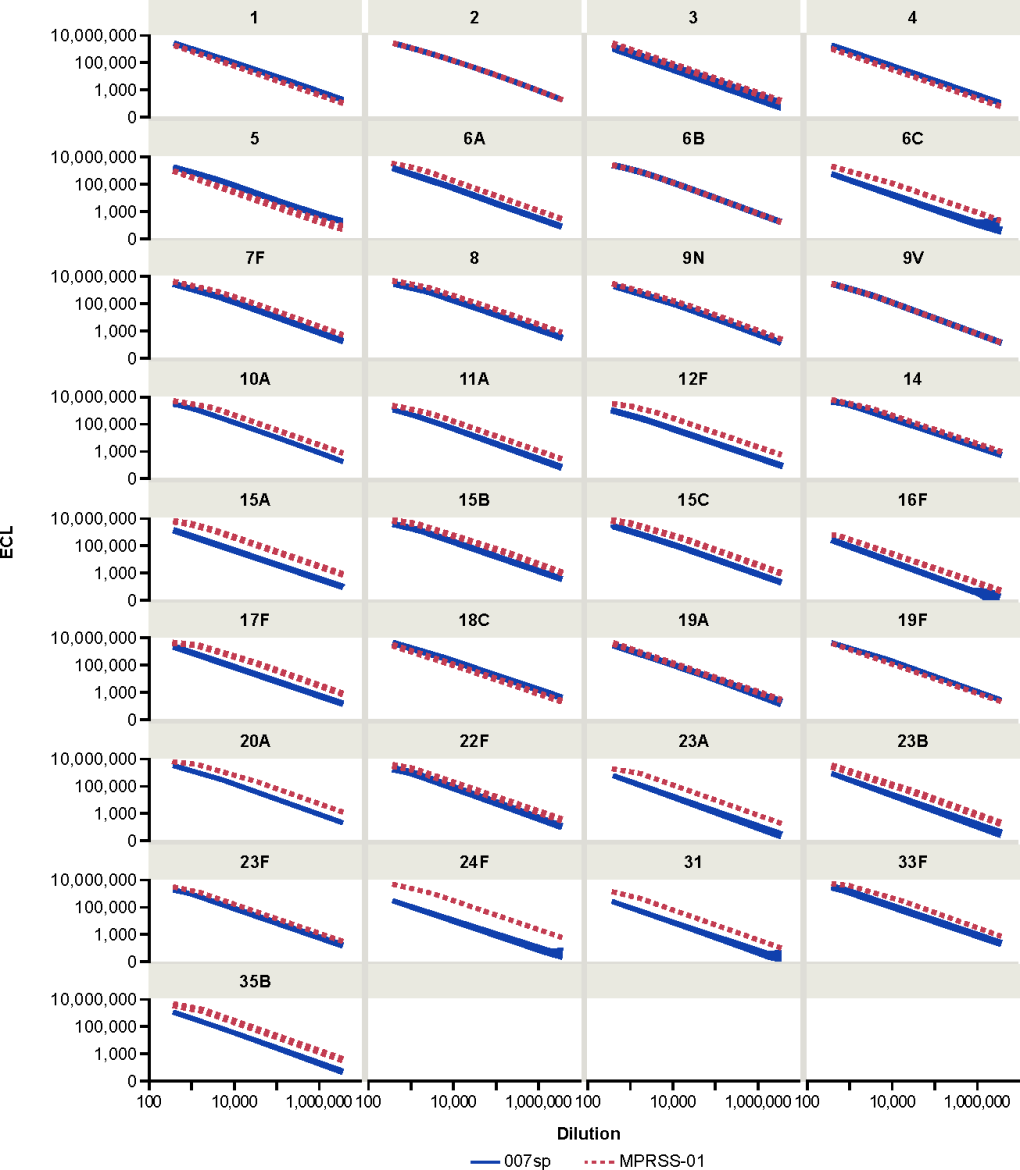
Fitted MPRSS-01 and 007sp dilution–response curves by serotype. ECL, electrochemiluminescence.

Given the similarity in slopes between MPRSS-01 and 007sp, a second model, in which a logistic regression function with common minimum, common maximum, and common slope parameters was applied to the data to estimate the calibration factor (i.e., the fold-difference in antibody concentration of the MPRSS-01 reference standard to the 007sp reference standard) relating the two reference standards. For the serotypes evaluated across more than one MSD plate, the calibration factor estimates were consistent across the differing MSD plate configurations, and, therefore, the estimates were combined across the runs and MSD plate configurations. The relative difference in anti-PnPs antibody concentration between the MPRSS-01 and 007sp reference standards is depicted graphically for each serotype in Fig. 3 via the difference in ECL signal when tested at the same dilution. The estimated calibration factor is provided for each of the 33 serotypes in Table 1. For each of the nine serotypes without a Goldblatt assignment, the anti-pneumococcal antibody concentration was appreciably higher in MPRSS-01 as compared with 007sp, ranging from 4.18-fold higher (serotype 16F) to 25.88-fold higher (serotype 24F). Table 1 also provides the estimated anti-pneumococcal antibody concentration for MPRSS-01 for each of the 24 serotypes with a 007sp concentration assignment.

### Assignment of antibody concentrations to MPRSS-01 for serotypes 6C, 15A, 16F, 23A, 23B, 24F, 31, 35B, and de-O-acetylated 15B (d15B) polysaccharide

The initial plan for assigning antibody concentrations to MPRSS-01 for serotypes 6C, 15A, 16F, 23A, 23B, 24F, 31, 35B, and d15B (henceforth referred to as 15C due to its structural similarity to 15C) was to calibrate each of the nine unique serotypes against serotype 7F within MPRSS-01 using the 20.24 µg/mL antibody concentration for serotype 7F obtained via direct calibration to 007sp. This standardization approach is analogous to the equivalence of absorbance method used by Quataert et al. (12) to provide 89SF concentration assignments for 13 serotypes relative to serotype 6B via serotype-specific ELISAs. For MPRSS-01, serotype 7F was chosen as the reference calibrator, as it was one of the original 89SF serotypes for which an antibody concentration was directly determined and because its assigned antibody concentration was close to the geometric mean concentration of the 24 assigned serotypes.

### Calibration to serotype 7F within 007sp

**Fig 4:**
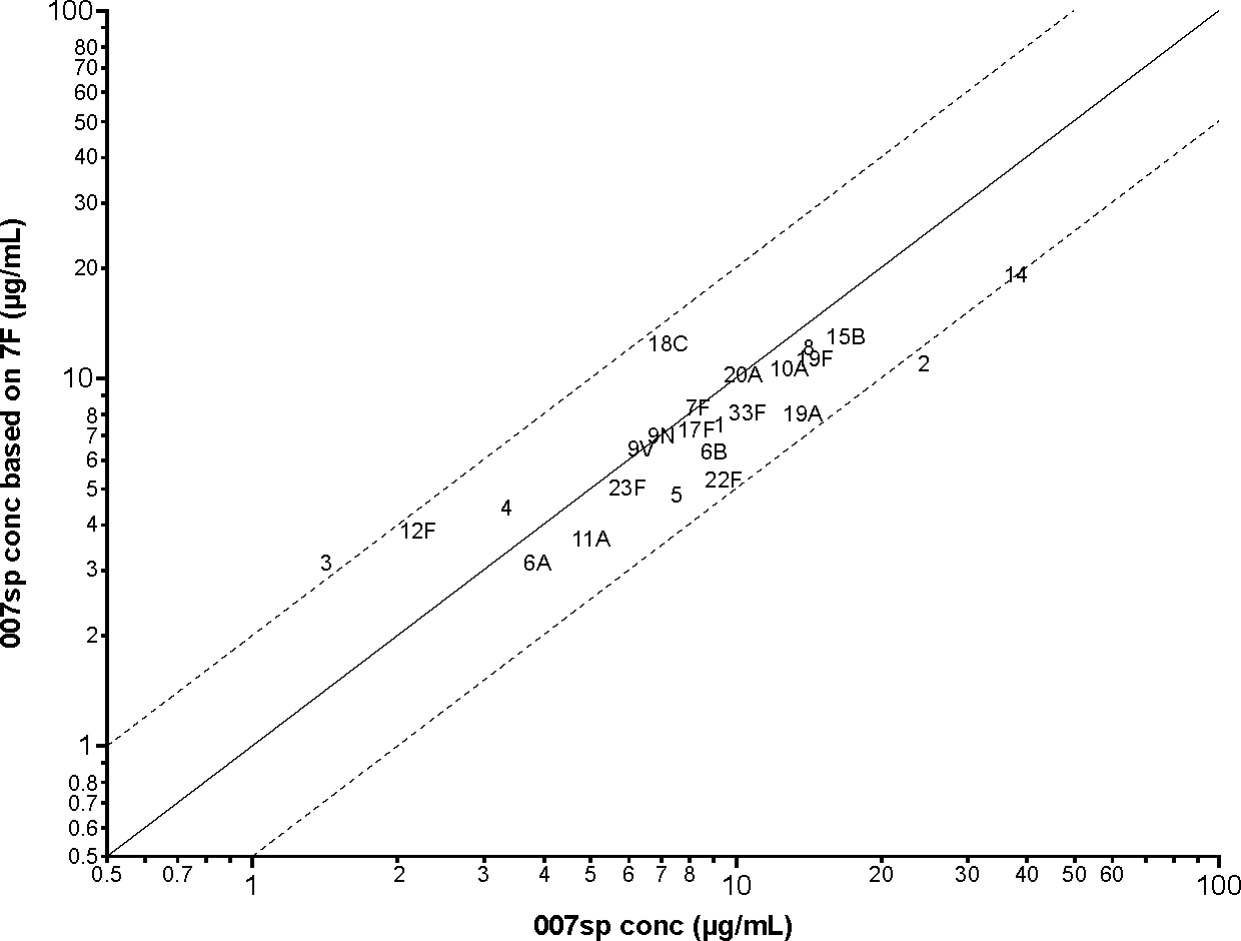
Comparison of the 007sp-estimated concentration based on calibration to 7F within 007sp to the Goldblatt-assigned concentration for the 24 Goldblatt-assigned serotypes. Solid line represents perfect agreement between the assignments; the dashed lines represent the ±2-fold bounds about the line of perfect agreement. Conc, concentration.

**Fig 5:**
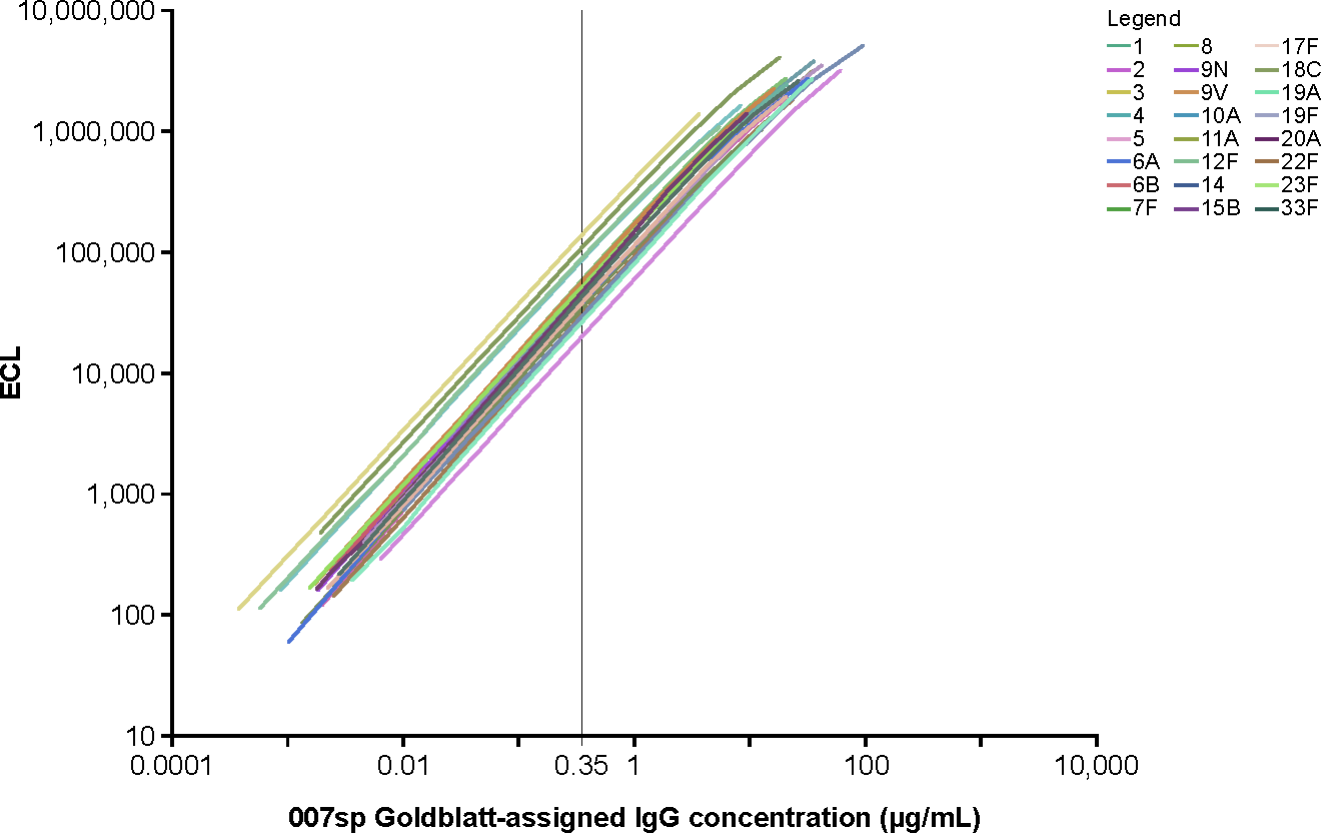
Individually fit 007sp concentration–response curves for the 24 Goldblatt-assigned serotypes. IgG concentrations are multiplied by 1,000 to reflect the Pn ECL minimum required test sample dilution of 1:1,000. IgG, immunoglobulin G; Pn ECL, pneumococcal electrochemiluminescence.

To assess the general applicability of calibrating against a single serotype to generate the antibody concentration assignments for the nine unassigned serotypes, antibody concentrations in 007sp were estimated for the 24 serotypes having a Goldblatt 007sp assignment via direct calibration to serotype 7F within 007sp. The accuracy of the approach was then assessed by comparing the resulting calibration estimates to their corresponding Goldblatt assignments. In theory, the antibody concentrations obtained by calibrating to serotype 7F should closely approximate the Goldblatt assignments. Parallel-line analysis using the four-parameter logistic regression function was used to estimate the calibration factor relating the dilution–response curve for each non-7F serotype to the dilution–response curve for serotype 7F. Each serotype was observed to dilute in parallel to 7F, as the slope ratios ranged from 0.95 (serotype 12F) to 1.02 (serotype 6A) across the 32 non-7F serotypes (Table 2). Parallel-line analysis using a common slope model was then used to obtain the calibration factors and antibody concentrations that are provided in Table 2. Table 2 also provides the antibody concentration ratio between the 7F calibration estimate and the Goldblatt-assigned value. Although the fold-difference between the estimated concentration and the assigned concentration was small for most serotypes, it exceeded twofold for serotypes 2, 3, and 14. Across the 24 serotypes having a Goldblatt-assigned concentration, the fold-difference between the estimated concentration and the assigned concentration ranged from 0.44 (serotype 2) to 2.17 (serotype 3), with a geometric mean concentration ratio (GMR) of 0.86. A graphical comparison between the estimated antibody concentrations based on calibration to serotype 7F within 007sp and the Goldblatt-assigned concentrations is provided in Fig. 4. The divergence between the estimated concentrations and the Goldblatt-assigned concentrations relates directly to what is graphically depicted in Fig. 5. Figure 5 shows the ECL signal plotted against the assigned antibody concentration for each of the 24 serotypes with a Goldblatt assignment. The 24 curves in Fig. 5 do not overlay one another. In fact, the horizontal shifts in the curves relative to serotype 7F align with the differences between the estimated and assigned concentrations for the 24 serotypes. Implications of the horizontal shifts in the curves are that: (i) the relative positioning of the 24 points in Fig. 4 is fixed, irrespective of the serotype selected for calibration, and, therefore, it is not possible to closely approximate the Goldblatt-assigned concentration for all 24 serotypes simultaneously using calibration methodology; and (ii) the range in MPRSS-01 concentration assignment could vary by as much 4.9-fold (i.e., 2.17/0.44), depending on the serotype against which the calibration is performed. For example, if the calibration was performed against serotype 2 rather than serotype 7F, each of the estimated antibody concentrations for 007sp in Table 2 would be increased by 2.27-fold (i.e., 1/0.44-fold), whereas if the calibration was performed against serotype 3, each of the estimated concentrations for 007sp in Table 2 would decrease by 2.17-fold. To remove the dependence on using a single serotype for the calibration, the strategy was revised to adjust the MPRSS-01 calibration estimates obtained via calibration to serotype 7F within MPRSS-01 by the corresponding GMR value of 0.86. In following this approach, the same set of concentration estimates for serotypes 6C, 15A, 15C, 16F, 23A, 23B, 24F, 31, and 35B would be obtained, irrespective of which of the 24 serotypes was chosen to be the calibrator.

**TABLE 2:** Calibration within 007sp and relative to 007sp serotype 7F^*a*^

Serotype	*N*	Average slope		Slope ratio (non-7F 007sp/7F 007sp)		Calibration factor (non-7F 007sp/7F 007sp)		007sp-assigned conc (µg/mL)	7F 007sp conc (µg/mL)	007sp-estimated conc based on 7F (µg/mL)		Ratio (007sp conc based on 7F 007sp/007sp conc)
		Non-7F 007sp	7F 007sp	Estimate	95% CI	Estimate	95% CI			Estimate	95% CI	
1	24	1.05	1.08	0.97	0.97–0.98	0.85	0.83–0.87	8.50	8.30	7.06	6.91–7.21	0.83
2	23	1.06	1.07	0.99	0.99–1.00	1.31	1.27–1.36	24.63	8.30	10.91	10.57–11.26	0.44
3	47	1.06	1.08	0.98	0.98–0.98	0.38	0.36–0.40	1.45	8.30	3.15	3.02–3.29	2.17
4	24	1.05	1.08	0.97	0.97–0.98	0.53	0.52–0.55	3.33	8.30	4.44	4.28–4.60	1.33
5	24	1.08	1.09	0.99	0.99–1.00	0.58	0.56–0.59	7.51	8.30	4.79	4.68–4.90	0.64
6A	47	1.11	1.09	1.02	1.01–1.03	0.38	0.36–0.39	3.93	8.30	3.12	3.01–3.23	0.79
6B	24	1.09	1.09	1.00	1.00–1.01	0.77	0.71–0.83	9.05	8.30	6.35	5.86–6.89	0.70
6C	23	1.11	1.09	1.02	1.01–1.03	0.17	0.16–0.17	ND	8.30	1.38	1.33–1.44	ND
7F	47	1.08	1.08	1.00	1.00–1.00	1.00	NA	8.30	8.30	8.30	NA	1.00
8	23	1.04	1.07	0.98	0.97–0.98	1.44	1.40–1.48	14.24	8.30	11.96	11.65–12.28	0.84
9N	23	1.09	1.08	1.00	0.99–1.01	0.82	0.80–0.84	7.03	8.30	6.83	6.65–7.01	0.97
9V	24	1.08	1.08	1.00	0.99–1.01	0.78	0.75–0.81	6.44	8.30	6.45	6.19–6.72	1.00
10A	23	1.08	1.08	1.00	1.00–1.01	1.28	1.25–1.31	12.98	8.30	10.62	10.36–10.89	0.82
11A	23	1.06	1.09	0.98	0.97–0.99	0.44	0.43–0.45	5.08	8.30	3.64	3.54–3.74	0.72
12F	23	1.03	1.09	0.95	0.94–0.95	0.47	0.45–0.48	2.21	8.30	3.87	3.77–3.98	1.75
14	24	1.05	1.07	0.98	0.98–0.99	2.26	2.19–2.34	37.99	8.30	18.77	18.19–19.38	0.49
15A	23	1.07	1.09	0.98	0.97–0.99	0.40	0.38–0.41	ND	8.30	3.28	3.15–3.42	ND
15B	35	1.07	1.08	0.99	0.99–1.00	1.55	1.50–1.60	16.94	8.30	12.85	12.44–13.29	0.76
15C	35	1.08	1.08	1.00	0.99–1.00	0.98	0.95–1.00	ND	8.30	8.09	7.90–8.29	ND
16F	23	1.11	1.08	1.02	1.01–1.03	0.07	0.07–0.07	ND	8.30	0.59	0.57–0.60	ND
17F	23	1.07	1.08	0.99	0.98–1.00	0.87	0.85–0.89	8.51	8.30	7.21	7.03–7.40	0.85
18C	24	1.06	1.07	0.99	0.98–0.99	1.50	1.43–1.57	7.30	8.30	12.43	11.87–13.02	1.70
19A	47	1.07	1.08	0.99	0.99–1.00	0.95	0.92–0.98	13.87	8.30	7.87	7.63–8.12	0.57
19F	24	1.07	1.07	1.00	0.99–1.00	1.36	1.32–1.40	14.61	8.30	11.29	10.95–11.65	0.77
20A	23	1.08	1.08	1.00	0.99–1.00	1.23	1.20–1.25	10.47	8.30	10.18	9.96–10.40	0.97
22F	47	1.07	1.08	0.99	0.99–0.99	0.63	0.62–0.65	9.50	8.30	5.25	5.13–5.38	0.55
23A	23	1.07	1.08	0.98	0.98–0.99	0.18	0.17–0.18	ND	8.30	1.49	1.45–1.53	ND
23B	23	1.10	1.09	1.01	1.01–1.02	0.24	0.23–0.25	ND	8.30	1.98	1.90–2.06	ND
23F	24	1.07	1.08	0.99	0.98–1.00	0.60	0.59–0.62	5.95	8.30	5.01	4.86–5.18	0.84
24F	23	1.05	1.08	0.97	0.96–0.97	0.10	0.10–0.11	ND	8.30	0.85	0.83–0.88	ND
31	23	1.09	1.08	1.01	1.00–1.02	0.08	0.08–0.09	ND	8.30	0.68	0.65–0.71	ND
33F	47	1.09	1.08	1.01	1.00–1.01	0.97	0.93–1.01	10.66	8.30	8.04	7.72–8.37	0.75
35B	23	1.11	1.09	1.02	1.01–1.03	0.36	0.35–0.38	ND	8.30	2.99	2.88–3.11	ND

^*a*^ Grey shading indicates the serotypes without previously published concentration assignments for 007sp. The slope ratio estimate and its 95% CI were derived from the individual slopes model, while the calibration factor and 007sp antibody concentration estimates were derived from the common slope model. International assignment does not exist. CI, confidence interval; Conc, concentration; NA, not applicable; ND, not determined.

### Calibration to serotype 7F within MPRSS-01

**Fig 6:**
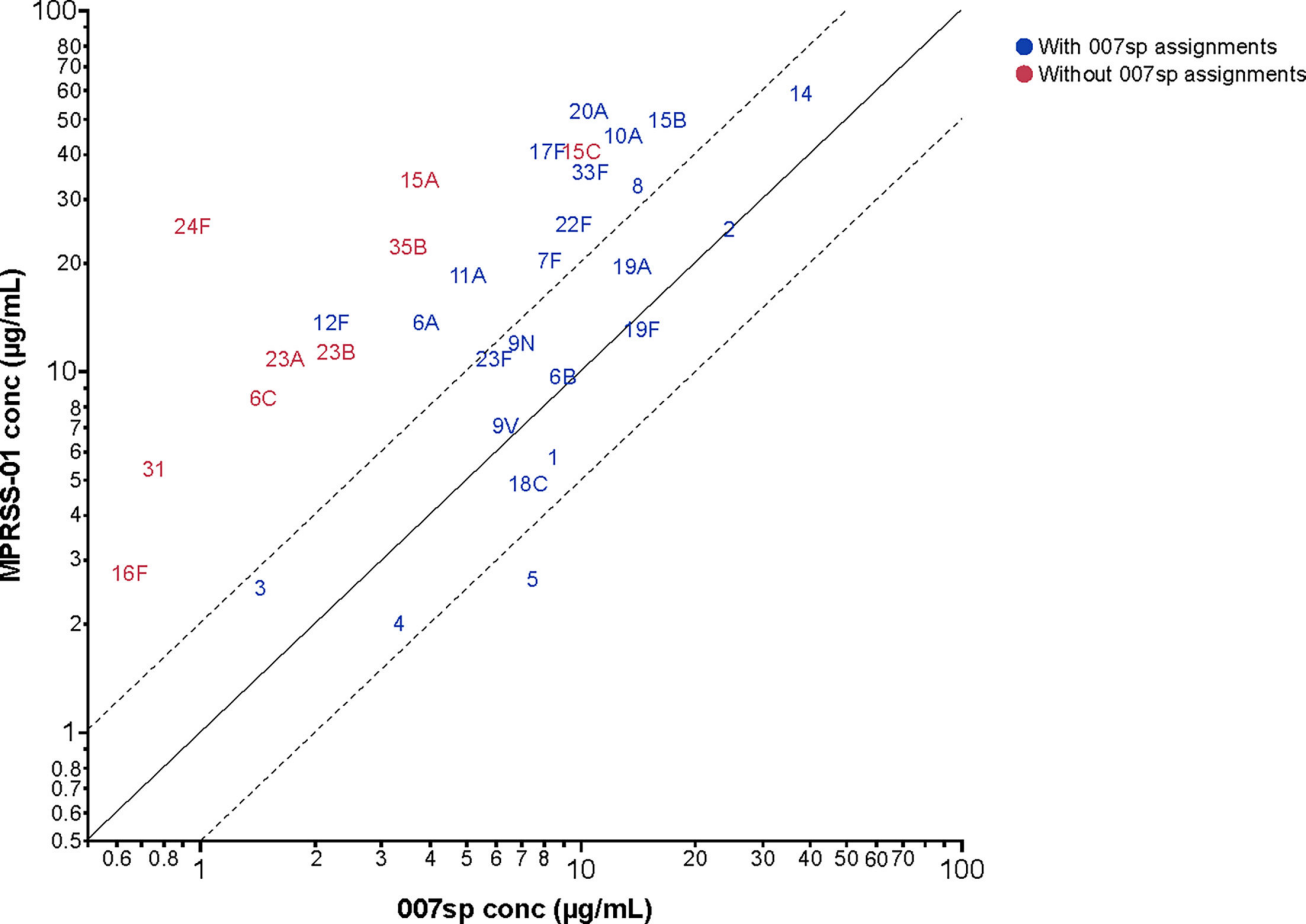
Concentration comparison between MPRSS-01 and 007sp. Solid line represents perfect agreement between the assignments; the dashed lines represent the ±2-fold bounds about the line of perfect agreement. Conc, concentration.

**Fig 7:**
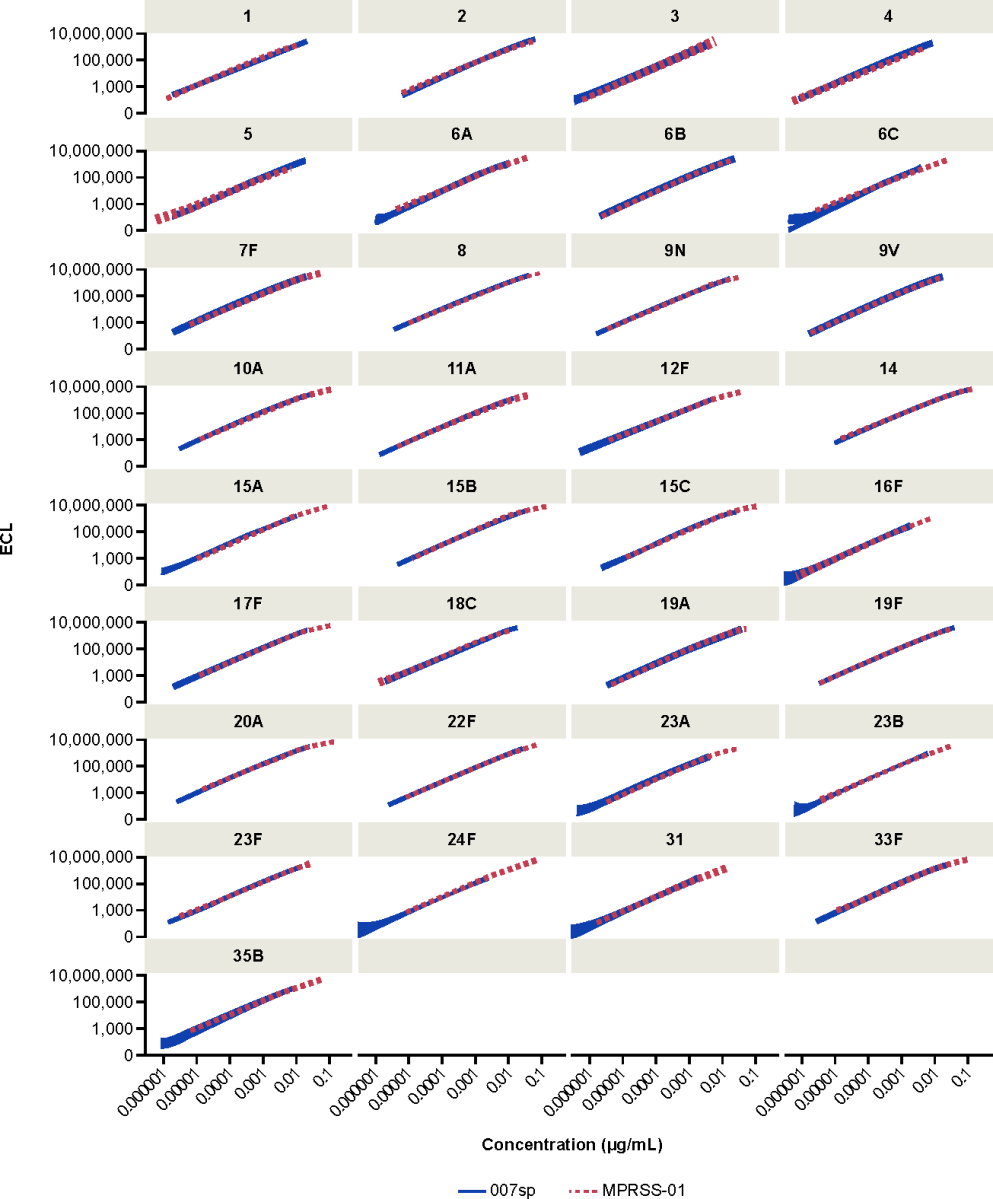
Fitted MPRSS-01 and 007sp concentration–response curves by serotype after application of the 007sp and MPRSS-01 concentration assignments. ECL, electrochemiluminescence.

In this assessment, each of the nine serotypes not having a Goldblatt-assigned concentration for 007sp (6C, 15A, 15C, 16F, 23A, 23B, 24F, 31, and 35B) was calibrated to serotype 7F within the MPRSS-01 reference standard using the MPRSS-01 concentration assignment of 20.24 µg/mL for 7F, arising from its direct calibration to 7F in 007sp. Parallel-line analysis using the four-parameter logistic regression function was used to estimate the calibration factor relating the dilution–response curve for each non-7F serotype to the dilution–response curve for serotype 7F. Each individual serotype was observed to dilute in parallel to 7F, as the slope ratios listed in Table 3 ranged from 0.97 to 1.03 across the nine serotypes. Parallel-line analysis using a common slope model was then used to obtain the calibration factors and antibody concentrations relative to serotype 7F that are provided in Table 3. The final concentration assignments in Table 3 were adjusted (divided) by the GMR value of 0.86, referenced in the previous section. Table 3 also provides estimated antibody concentration assignments for 007sp for serotypes 6C, 15A, 15C, 16F, 23A, 23B, 24F, 31, and 35B. Those assignments were determined using the final MPRSS-01 antibody concentration assignments and the calibration factor relating MPRSS-01 to 007sp for those serotypes. A comparison of all final MPRSS-01 concentration assignments to the corresponding 007sp concentration assignments is provided graphically in Fig. 6. The MPRSS-01 and 007sp standard curve profiles after application of the final concentration assignments are provided in Fig. 7. The overlaying of the MPRSS-01 and 007sp curves for each serotype supports the accuracy of the MPRSS-01 antibody concentration assignments relative to 007sp.

**TABLE 3:** Calibration within MPRSS-01 and relative to MPRSS-01 serotype 7F^*a*^

Serotype	*N*	Average slope		Slope ratio (MPRSS-01/7F MPRSS-01)		Calibration factor (MPRSS-01/7F MPRSS-01)		7F MPRSS-01 conc (µg/mL)	MPRSS-01 conc based on 7F MPRSS-01 (µg/mL)		GMR-adjusted antibody conc (µg/mL)^*b*^			
		MPRSS-01	7F MPRSS-01	Estimate	95% CI	Estimate	95% CI		Estimate	95% CI	MPRSS-01		007sp	
											Estimate	95% CI	Estimate	95% CI
6C	23	1.01	1.04	0.97	0.97–0.98	0.35	0.34–0.37	20.24	7.16	6.82–7.52	**8.34**	7.94–8.76	**1.48**	1.41–1.56
15A	23	1.07	1.04	1.03	1.03–1.04	1.42	1.36–1.47	20.24	28.66	27.62–29.75	**33.38**	32.17–34.65	**3.82**	3.68–3.96
15C	35	1.06	1.04	1.02	1.02–1.03	1.71	1.67–1.75	20.24	34.58	33.71–35.48	**40.28**	39.27–41.33	**9.50**	9.26–9.75
16F	23	1.08	1.04	1.03	1.02–1.04	0.12	0.11–0.12	20.24	2.34	2.26–2.41	**2.73**	2.63–2.81	**0.65**	0.63–0.67
23A	23	1.08	1.06	1.02	1.01–1.03	0.45	0.44–0.46	20.24	9.15	8.96–9.35	**10.66**	10.44–10.89	**1.69**	1.65–1.72
23B	23	1.07	1.04	1.03	1.02–1.04	0.47	0.45–0.50	20.24	9.59	9.06–10.15	**11.17**	10.55–11.82	**2.29**	2.16–2.42
24F	23	1.04	1.04	1.00	1.00–1.01	1.06	1.02–1.09	20.24	21.39	20.70–22.12	**24.92**	24.11–25.77	**0.96**	0.93–1.00
31	23	1.07	1.05	1.03	1.02–1.03	0.23	0.21–0.24	20.24	4.56	4.32–4.82	**5.31**	5.03–5.61	**0.76**	0.72–0.80
35B	23	1.07	1.04	1.03	1.02–1.03	0.93	0.88–0.99	20.24	18.89	17.83–20.00	**22.00**	20.77–23.3	**3.53**	3.33–3.74

^*a*^ Bolded values indicate MPRSS-01 and 007sp final concentration assignments for the nine serotypes not having a Goldblatt-assigned 007sp antibody concentration. CI, confidence interval; Conc, concentration; GMR, geometric mean concentration ratio.

^*b*^ Concentrations are adjusted using the value of 0.86, the geometric mean fold-ratio between the calibrated concentration and the established concentration determined across the 24 serotypes having established concentrations.

## DISCUSSION

With more than 100 pneumococcal serotypes in circulation, new PCVs containing serotypes to address the changing pneumococcal serotype epidemiological landscape are needed. To support future PCVs, an unmet need for a new *S. pneumoniae* reference serum standard containing serotypes beyond the 24 assigned in 007sp is recognized. Here, we describe the development of a new pneumococcal reference standard, MPRSS-01, with serotype-specific IgG concentrations for the 24 serotypes contained in 007sp, and nine novel serotypes. Creating a new pneumococcal reference serum and retro calibrating the 007sp for novel serotypes are crucial to support ongoing assessments of next-generation pneumococcal vaccines. The new standard will also help preserve the connection with the initial serological evaluations conducted before licensure in pivotal efficacy studies. In addition to evaluating immune responses to vaccines, the new reference standard could be used in research studies of natural immune responses to a broader range of pneumococcal serotypes in immunology studies (13).

For each of the 24 serotypes having a 007sp-established IgG concentration, the corresponding antibody concentration in MPRSS-01 was assigned via direct calibration to 007sp. For the nine novel serotypes not having a 007sp-established IgG concentration, a cross-standardization approach was undertaken. The calibration study demonstrated parallelism of the dilution–response curves across the 33 serotypes, both within and between the MPRSS-01 and 007sp reference standards, permitting the choice of any of the 007sp-assigned serotypes as the reference calibrator. Serotype 7F was chosen as the reference calibrator as it was one of the original serotypes for which an antibody concentration was directly determined and because its assigned antibody concentration was close to the geometric mean concentration of the 24 assigned types. The use of the single-calibrator approach was assessed by comparing the Goldblatt-assigned 007sp IgG concentration for each of the 24 serotypes to the concentration obtained by calibrating each serotype to 7F. Although the observed difference for most serotypes was small, there were a few serotypes for which the difference exceeded twofold. Although the relative differences in concentration assignment among the serotypes were preserved, independent of the choice of calibrator, this raised the possibility that systematic bias could be introduced by the choice of calibrator. For example, if serotype 19A was chosen for the calibration instead of 7F, the relative differences among the serotypes would be preserved, but the concentration assignments would be increased by a factor of 1.75-fold relative to the assignments obtained by calibrating against serotype 7F. Alternatively, if serotype 12F was chosen for the calibration instead of 7F, the relative differences among the serotypes would be preserved, but the concentration assignments would be decreased by a factor of 1.75-fold (i.e., 1/0.57-fold) relative to the assignments obtained by calibrating against serotype 7F. To address this concern, the estimated IgG concentrations of the nine novel serotypes were further adjusted using the geometric mean fold ratio between the calibrated concentration and the established concentration determined across the 24 serotypes. The adjustment by the GMR provides an alternative and near-equivalent method of cross-standardization to that performed by Concepcion and Frasch to estimate antibody concentrations in 89SF (14), in which each serotype is calibrated against every other serotype and the resulting geometric mean across the serotypes is the determined concentration. For MPRSS-01, application of the GMR adjustment provided a 24-fold reduction in the number of pairwise calibrations performed relative to the number required following the cross-standardization approach of Concepcion and Frasch (14). Across the 24 serotypes, the geometric mean fold difference between the GMR-adjusted 7F-calibrated concentrations and their corresponding Goldblatt assignments was 1.00. As the dilution–response curves were parallel both within and between the MPRSS-01 reference standards across the 33 serotypes, the above approach would result in near-equivalent GMR-adjusted concentration estimates for any of the 24 serotypes chosen as the calibrator. Thus, the GMR adjustment renders the final calibration estimates independent of the selected serotype.

As noted in the above discussion, the potential for systematic bias due to the choice of serotype chosen for calibration relates back to the inability of the concentration–response curves to overlay one another in the Pn ECL format. While the relative shifts among the curves could be due to differences in pre-adsorbents or antigen coating concentration between the Pn ECL and ELISA formats, it is also possible that such differences among the serotypes arose from the differences in format among the individual ELISAs performed at the time of the initial antibody concentration assignment. Originally, anti-PnPs antibody levels were assigned to the anti-pneumococcal standard serum, Lot 89SF, by assigning the concentration of known total Ig for reference preparation USNRP IS 1644 giving an absorbance of 0.3 in the concurrently run reference ELISA to the dilution of 89SF yielding an absorbance of 0.3 in the corresponding anti-PnPs-specific assay for total Ig (2). The dilution of 89SF yielding an absorbance of 0.3 within a specific anti-PnPs assay was likely dependent on the ELISA format carried out at that time. For example, a twofold higher PnPs coating concentration could increase the signal in the ELISA such that an absorbance of 0.3 might be achieved at a twofold lower dilution of 89SF. Individual coating concentrations are among the differences across ELISAs that could have differentially influenced the magnitude of the absorbances and, hence, the original antibody assignments. Quataert et al. (2) used coating concentrations for the antigens diluted in sterile phosphate-buffered saline on Nunc Microwell plates (catalog no. 2-69620) at 0.5 µg/mL for PnPs serotypes 4 and 9V; 1 µg/mL for PnPs serotypes 7F, 14, and 18C; 2 µg/mL for PnPs serotype 1; 5 µg/mL for PnPs serotype 5; and 10 µg/mL for PnPs serotypes 3, 6B, 19F, and 23F. In addition, the differences in the pre-adsorbents used when the concentrations were assigned to 89SF and 007sp could have had a role in the inability of the curves to overlay one another. That is, the original assignments to 89SF by Quataert et al. (2, 12) were done only with CWPS, whereas the values derived from the 007sp calibration by Goldblatt et al. (3, 4) followed double adsorption of 007sp with CWPS and heterologous polysaccharide 22F or mono- and di-phosphocholine-substituted CWPS to further remove the non-type-specific antibodies from sera. Serum pre-adsorption for the Pn ECL assay used in our study was performed using a different methodology (CWPS + PnPs 25 + PnPs 72) than Goldblatt et al. to improve assay specificity. This differs from the WHO reference ELISA, which uses CWPS + 22F, rather than the CWPS multicomplex (15). A previous study has shown similar results using both methods; thus, this was unlikely to affect our findings (16).

Despite the limitations of the calibration method noted above, many of the differences in reported antibody concentrations of test samples due to differences in assay format can be minimized using a common reference standard. For example, consistency between the Pn ECL and WHO ELISA, and concordance at the 0.35 µg/mL threshold for each serotype in V114, has been demonstrated when 007sp was used as the reference standard and the Goldblatt assignments were applied (7).

Although beyond the scope of this paper, an alternative approach would be to assess the anti-pneumococcal antibody concentration of the nine novel serotypes in 007sp, based on a relevant bioanalytical measure that is independent of the calibration approach. While such an approach, if applied to all serotypes, could perhaps address the potential inaccuracies in the 007sp assignments for serotypes 2 and 3, at the practical level, all pneumococcal clinical immunogenicity, as measured in binding assays, is tied to the concentration assignments in 89SF and 007sp. As such, the current concentration assignments constitute the historical frame of reference and, therefore, any deviation from that would need to be weighed against the added regulatory risk associated with a change in 007sp assignment.

**Fig 8:**
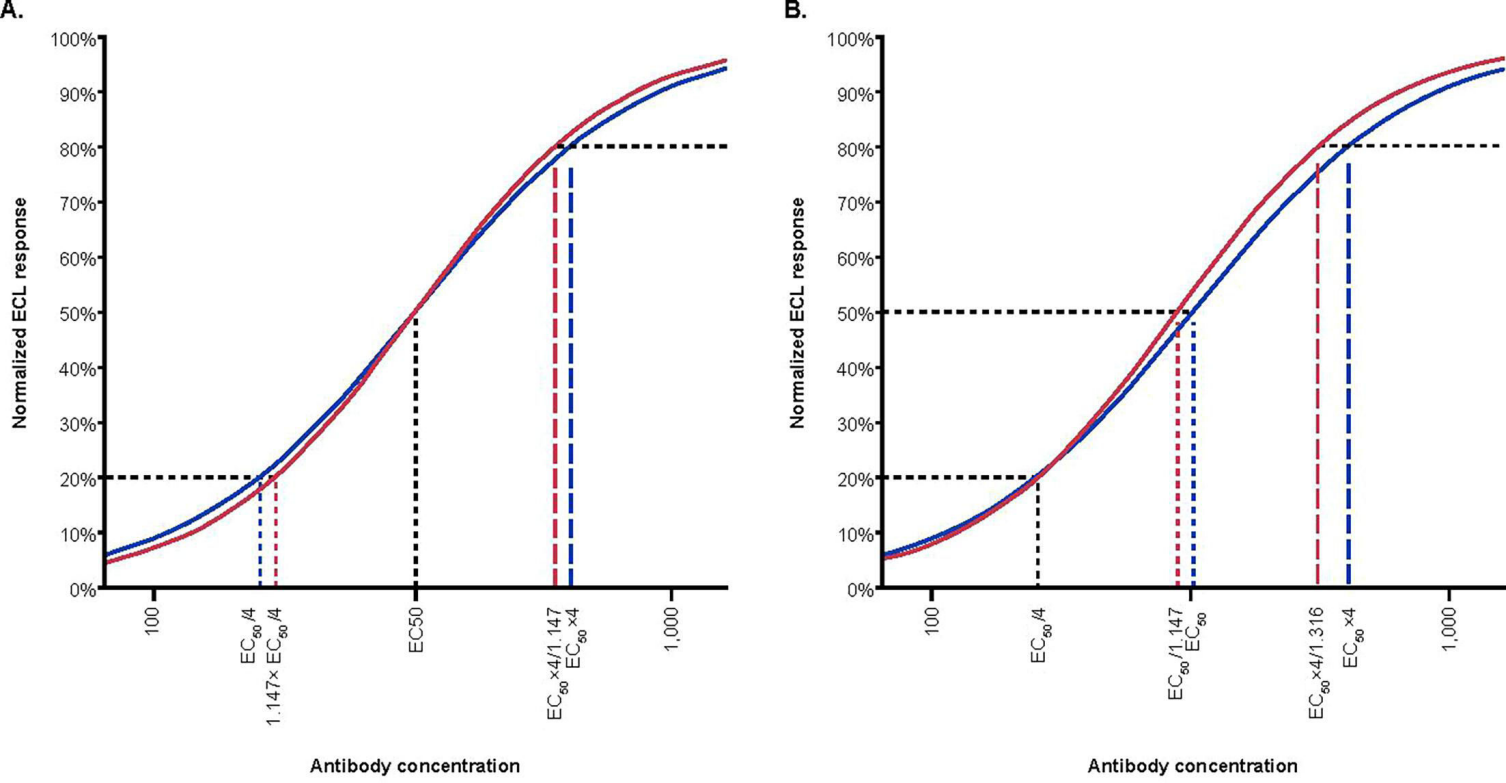
Illustrative example of the impact of the effect of reference standard non-parallelism on back-calculated antibody concentration of an unknown test sample. ECL, electrochemiluminescence; EC_50_, median effective concentration; RS, reference standard.

Although the approach of simultaneously fitting the four-parameter logistic function to the MPRSS-01 and 007sp dilution–response curves and assessing MPRSS-01 antibody concentrations based on the ratio of the median effective dilution (ED_50_) parameters and parallelism based on the ratio of the slopes from the four-parameter logistic regression fits was used, the determinations of MPRSS-01 antibody concentrations and parallelism to 007sp could have been accomplished by back-calibrating the MPRSS-01 dilution series against the fitted 007sp reference standard curve. In fact, the Goldblatt concentration assignments for 007sp were obtained by back-calibrating 007sp against 89SF. In our experience, both approaches are valid and, if properly performed, will result in similar estimates and conclusions. In addition, the back-calibrated concentrations allow for an assessment of parallelism between the reference standards. Regarding the slope ratio from the four-parameter fit, the impact of non-parallelism can be expressed in terms of its effect on the back-calibrated antibody concentration for an unknown test sample. For example, assuming an ED_50_ ratio of 1.00 and slope estimates of 1.00 for 007sp and 1.11 for MPRSS-01, arising from the fit of an individual slopes model of the form described in this paper, then, relative to the current reference standard, the new reference standard would result in an overestimate of the antibody concentration in an unknown sample of 1.147-fold at the 20th percentile of the response distribution, an equivalent estimate of antibody concentration at the 50th percentile, and an underestimate of antibody concentration of 1.147-fold at the 80th percentile of the response distribution (Fig. 8A). Given the magnitude of that effect on antibody concentration, the acceptable range for the slope ratio was set to 0.90–1.11. In general, the degree of non-parallelism that is considered acceptable depends on the assay and the context in which it is used. If the magnitude of the non-parallelism is judged to be impactful, the calibration factor can be chosen such that the two reference standards yield identical back-calibrated concentrations at a particular concentration, such as a designated antibody cut point. However, that approach incurs a trade-off, in that it will result in larger differences in back-calculated antibody concentrations between the two reference standards in other regions of the quantifiable range. Such a trade-off is graphically illustrated in Fig. 8B, in which a calibration factor of 1.147 is applied to the new reference standard given in Fig. 8A. Relative to the current reference standard, the new reference standard would result in an equivalent estimate of antibody concentration at the 20th percentile of the response distribution, an underestimate of the antibody concentration of 1.147-fold at the 50th percentile of the response distribution, and an underestimate of antibody concentration of 1.316-fold at the 80th percentile of the response distribution.

The MPRSS-01 reference standard was implemented for Phase III clinical testing of V116. Moreover, the formal calibration of MPRSS-01 was accepted as a new human pneumococcal standard reference serum by the Center for Biologics Evaluation and Research (CBER), signifying that it has met stringent regulatory requirements, undergone rigorous validation and quality control, and can be trusted for accurate and reproducible measurements (17). We have also developed an IgG assignment calibration platform that can be leveraged to develop new reference standards to support PCVs with novel serotypes. To conclude, utilizing this new reference standard will help support V116 and potential future PCVs to maintain the link to the historical human pneumococcal standard reference serum while adopting the new human pneumococcal reference serum.

## APPENDIX

Define the slope ratio as $\frac{{\hat{B}}_{1j}}{{\hat{B}}_{2j}}$, in which ${\hat{B}}_{1j}$ and ${\hat{B}}_{2j}$ are the slope estimates from the *j*^th^ run of a particular curve pairing, *j* = 1, 2, …, *n*. The approximate variance of the natural logarithm of the slope ratio, that is ${\hat{M}}_{j}=ln\left(\frac{{\hat{B}}_{1j}}{{\hat{B}}_{2j}}\right)$, is obtained via the delta method (18) as:


$$Var\left({\hat{M}}_{j}\right)=\frac{Var\left({\hat{B}}_{1j}\right)}{{{\hat{B}}_{1j}}^{2}}+\frac{Var\left({\hat{B}}_{2j}\right)}{{{\hat{B}}_{2j}}^{2}}-\frac{2\times Cov\left({\hat{B}}_{1j},{\hat{B}}_{2j}\right)}{{\hat{B}}_{1j}\times {\hat{B}}_{2j}}$$

An overall estimate of the slope ratio for each serotype is given by $e^{{\bar{M}}_{w}}$, in which ${\bar{M}}_{w}=\sum_{j=1}^{n}\left(w_{j}\times {\hat{M}}_{j}\right)/\sum_{j=1}^{n}\left(w_{j}\right)$ denotes the weighted average of the individual ${\hat{M}}_{j}$ estimates. The *j*^th^ weight, $w_{j}$, is the reciprocal of the sum of the variance of the *j*^th^ slope ratio estimate and the variance among the *n* estimates that is common to all estimates. That is, $w_{j}=\frac{1}{Var\left({\hat{M}}_{j}\right)+Var\left(m\right)}$, in which $Var\left(m\right)=\;\frac{\sum_{j=1}^{n}\left({\hat{M}}_{j}^{2}\right)-\frac{{\left(\sum_{j=1}^{n}{\hat{M}}_{j}\right)}^{2}}{n}}{n-1}-\;\frac{\sum_{j=1}^{n}\left[Var\left({\hat{M}}_{j}\right)\right]}{n}$. The variance of the weighted average is given by $Var\left({\bar{M}}_{w}\right)={\left(\sum_{j=1}^{n}w_{j}\right)}^{-1}$, and the $\left(1-\propto /2\right)\times 100\%$ confidence interval about ${\bar{M}}_{w}$ is given by ${\bar{M}}_{w}\pm t_{\propto /2,\;n-1}\times \sqrt{Var\left({\bar{M}}_{w}\right)}$. The corresponding back-transformed CI for the overall slope ratio estimate is $e^{{\bar{M}}_{w}\pm t_{\propto /2,\;n-1}\times \sqrt{Var\left({\bar{M}}_{w}\right)}}$.

The calculations for the overall calibration factor and its 95% CI for each curve pairing follow those for the slope ratio, but with ${\hat{C}}_{1j}$, ${\hat{C}}_{2j}$ and their corresponding variance estimates replacing ${\hat{B}}_{1j}$, ${\hat{B}}_{2j}$ and their corresponding variance estimates, respectively.

## Data Availability

The data sharing policy, including restrictions, of Merck Sharp & Dohme LLC, a subsidiary of Merck & Co., Inc., Rahway, NJ, USA (MSD), is available at http://engagezone.msd.com/ds_documentation.php. Requests for access to the clinical study data can be submitted through the Engage Zone site or via email to dataaccess@msd.com.
